# Prevalence and related factors of anxiety symptoms in patients with major depressive disorder and a history of recent suicide attempts: cross-sectional study

**DOI:** 10.1192/bjo.2026.11000

**Published:** 2026-03-30

**Authors:** Quanfeng Zhu, Wenli Chen, Xiaoe Lang, Yali Zheng, Xiang-Yang Zhang

**Affiliations:** Department of Psychiatry, Affiliated Xiaoshan Hospital, Hangzhou Normal University, Hangzhou, China; Department of Psychiatry, First Hospital/First Clinical Medical College of Shanxi Medical University, Taiyuan, China; Affiliated Mental Health Center of Anhui Medical University; Hefei Fourth People’s Hospital; Anhui Mental Health Center, Hefei, China

**Keywords:** Major depressive disorder, anxiety, recent suicide attempts, positive psychotic symptoms, thyroid function

## Abstract

**Background:**

Patients with major depressive disorder (MDD) are known to be at a heightened risk of suicide. Research indicates that comorbid anxiety may further elevate this risk. However, studies specifically examining the prevalence of anxiety and its associated factors among patients with MDD and history of a recent suicide attempt (RSA) remain limited.

**Aims:**

To investigate the prevalence of anxiety and the factors associated with its severity in patients with MDD.

**Method:**

A total of 1718 patients with MDD were included. Hypothesis testing and binary logistic regression were employed to examine differences in anxiety severity and clinical factors between patients with and without an RSA, as well as the association between anxiety severity and RSA. Subsequently, patients with MDD were categorised into three subgroups based on anxiety severity. One-way analysis of variance and multivariate logistic regression were then conducted to identify factors associated with anxiety symptoms.

**Results:**

Anxiety severity was identified as an independent correlate of RSA. Compared with patients with anxiety, patients with MDD and significant or severe anxiety had a 2.9-fold and 11.8-fold increased risk of RSA, respectively. Furthermore, Hamilton Rating Scale for Depression score, Positive and Negative Syndrome Scale positive subscale score, and thyroid function indices (free triiodothyronine and thyroid-stimulating hormone levels) were also determined to be independent correlates of anxiety severity.

**Conclusions:**

Anxiety is associated with risk of RSA in patients with MDD, and depressive symptoms, positive psychotic symptoms and thyroid function may be related factors for severity of anxiety.

Major depressive disorder (MDD) is a serious and common mental illness that takes a physical and psychological toll. Individuals with MDD face a significantly higher risk of suicide compared with the general healthy population. Evidence suggests that in patients with depression, those with suicidal ideation or behaviour tend to exhibit more severe depressive symptoms and a greater burden of psychiatric comorbidities.^1^ Furthermore, comorbid anxiety is also recognised as a predictor of suicide attempts and suicidal behaviour in patients with MDD.^2^ The proportion of patients with MDD and comorbid anxiety is high, and anxiety can affect attention and cognitive function.^3^ Interestingly, it has been suggested that depression comorbid with anxiety constitutes a distinct clinical entity, whose impact on cognitive function differs from that of either condition alone.^4^ Findings in this regard have been inconsistent, with Basso et al finding attention and executive dysfunction and psychomotor slowing in patients with MDD and comorbid anxiety.^5^ In contrast, Castaneda et al found that comorbid anxiety did not alter any of the neuropsychological variables, including attention, in patients with MDD.^6^

Evidence indicates a complex link between suicide risk in MDD and metabolic and endocrine measures. Mendelian randomisation analyses suggest a potential positive causal role of elevated triglycerides in depressive symptoms and self-harm, with a moderate association observed for high-density lipoprotein cholesterol (HDL-C).^7^ Clinical studies further reveal a profile of lipid dysregulation and mitochondrial dysfunction in treatment-resistant MDD with suicidal ideation.^8^ Findings on lipids and recent suicidal behaviour, however, are inconsistent, with reports of both lower triglycerides and higher very-low-density lipoprotein in relation to suicide attempts.^9^ Additionally, thyroid dysfunction is implicated, as higher levels of thyroid-stimulating hormone (TSH) and low-density lipoprotein cholesterol (LDL-C) correlate with suicide attempts in adolescents, and are linked to altered brain dynamics.^10,11^ Although lipid and thyroid parameters appear connected to suicide risk in MDD, the direction and mechanisms of these associations remain unclear and are likely modulated by clinical and individual factors, necessitating further research to clarify their pathological significance.

Although anxiety symptoms have been widely recognised as significantly associated with suicide risk in patients with MDD, the correlation between different levels of anxiety severity – stratified using the internationally validated Hamilton Rating Scale for Anxiety (HRSA) – and suicide risk remains unclear. Furthermore, the relationship between metabolic factors, such as glucose-lipid levels and thyroid function, and the severity of anxiety in patients with MDD and history of recent suicide attempts (RSA) also requires further clarification. Existing studies have predominantly treated anxiety as a binary variable or analysed anxiety scale scores as a continuous measure. By categorising anxiety severity into three levels based on HRSA scores, this study allows for a more sensitive detection of the continuous gradient of anxiety symptoms in patients with MDD. This approach facilitates the examination of potential non-linear relationships between different anxiety levels and suicide risk or clinical features, thereby providing empirical evidence for clinically meaningful thresholds that reflect a transition from quantitative to qualitative change. Methodologically, this enables a more refined characterisation of anxiety in MDD. Theoretically, it deepens the understanding of heterogeneity within the MDD population at risk for suicide. Clinically, it offers evidence to support personalised intervention strategies. Thus, the present study addresses a significant gap in systematically investigating the severity dimension of anxiety in high-risk MDD populations, demonstrating clear innovative and translational potential.

## Method

### Participant recruitment

A total of 1718 eligible participants were enrolled from the First Hospital of Shanxi Medical University. The inclusion criteria were as follows: (a) aged 18–65 years, (b) meeting the DSM-Ⅳ diagnostic criteria for MDD and (c) first-episode and drug-naïve status. Exclusion criteria comprised (a) any comorbid Axis 1 psychiatric disorder other than MDD, (b) severe physical illness, (c) history of substance or drug dependence and (d) pregnancy or lactation.

### Information collection and biochemical index detection

The gender, age, body mass index (BMI), education, marital status and disease duration of each participant were collected, and diastolic blood pressure (DBP) and systolic blood pressure (SBP) were measured. In addition, participants were instructed to refrain from eating after 20.00 h and drinking after 22.00 h, and venous blood was collected from the median cubital vein before 08.00 h the following morning. The collected blood sample was immediately sent to the hospital laboratory department and tested within 1 h. The blood indexes detected in this study included fasting blood glucose (FBG), total serum cholesterol, triglyceride, LDL-C, HDL-C, free triiodothyronine (FT3), free thyroxine, TSH, anti-thyroglobulin antibody (TgAb) and thyroid peroxidase antibody (TPOAb). In this study, abnormal TSH, TgAb and TPOAb were defined as TSH >4.2 mIU/L, TgAb >115 IU/L and TPOAb >34 IU/L.

### Assessment of clinical symptoms

The 17-item Hamilton Rating Scale for Depression (HRSD), 14-item HRSA and Positive and Negative Syndrome Scale (PANSS) positive subscale were used to assess the depression, anxiety and psychosis positive symptoms of participants, respectively. The Chinese versions of these scales have been demonstrated to have good reliability and validity in the Chinese population.^12–14^ The scale assessments performed on each participant were completed by staff with standardised training. Based on extensive clinical experience, standards from pharmacological clinical trials and the general consensus within the academic community, this study adopted the HRSA cut-off values most appropriate for the Han Chinese population to categorise anxiety symptoms in patients with MDD into three levels: an HRSA score of 14 to < 21 was defined as mild anxiety, 21 to < 29 as significant anxiety and ≥29 as severe anxiety.^15^

### Investigation of RSA

The RSA status was investigated through simultaneous interviews with the participants and their family members. Each participant and family member were asked: Has the participant attempted suicide in the past month? If the answer was ‘yes’, they were then asked how many times, how and when.

### Study protocol and statistical methods

**Fig. 1:**
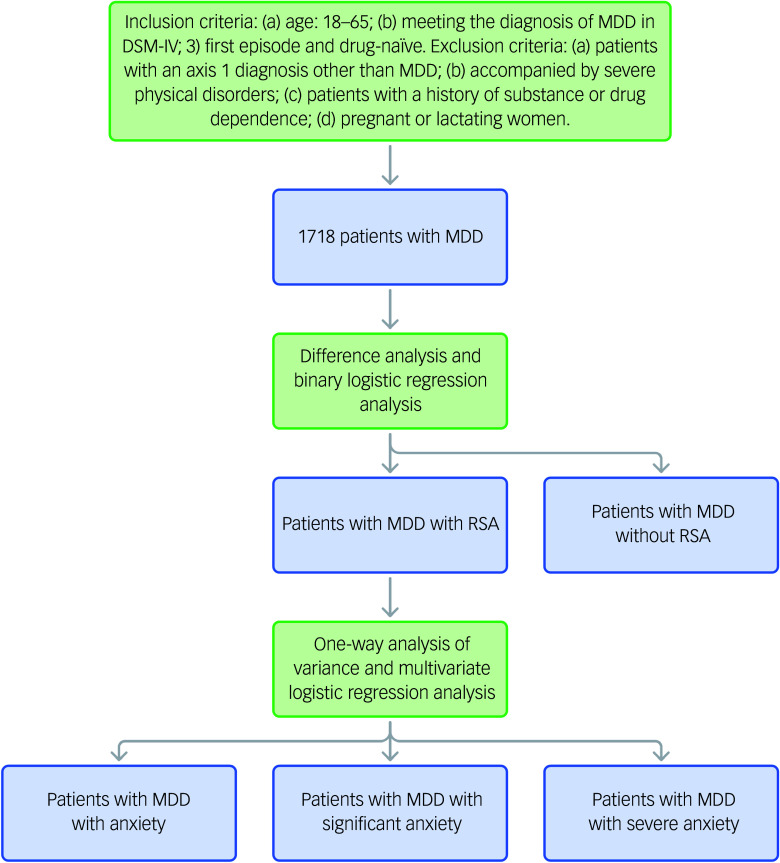
Study flowchart. MDD, major depressive disorder; RSA, recent suicide attempts.

The flowchart of this study is presented in Fig. 1. Following an analytical pathway that included group comparisons, regression modelling and stratified refinement, and employing both parametric and non-parametric methods, we incorporated resampling techniques and multiple corrections to enhance the robustness and reliability of the results. This approach progressively elucidated the association between RSA and anxiety severity, as well as its influencing factors. All statistical analyses in this study were performed using IBM SPSS Statistics (version 25.0). First, this study investigated the prevalence of RSA in 1718 patients with MDD and divided the patients into two subgroups: those with an RSA and those without an RSA. Differences in anxiety severity and other variables – including sociodemographic information, biochemical indicators and clinical symptoms – between the two subgroups were analysed using hypothesis testing. The impact of anxiety severity on RSA was examined by binary logistic regression analysis. Second, among patients with MDD and a history of RSA, we further stratified them into three subgroups based on anxiety levels – anxiety, significant anxiety and severe anxiety. One-way analysis of variance (ANOVA) and multivariate logistic regression were conducted to analyse differences in other clinical factors across these subgroups and to identify independent risk factors associated with varying levels of anxiety severity.

In the hypothesis testing between the two groups, normality was assessed with the Kolmogorov–Smirnov test. Continuous variables conforming to a normal distribution were analysed with the *t*-test, and those not conforming to a normal distribution were analysed with the Mann–Whitney *U*-test. Categorical variables were analysed with the chi-squared test. For comparisons of continuous variables among three groups, ANOVA was applied. To mitigate the influence of non-normal distribution and heteroscedasticity of the independent variables on the results, a bootstrap sampling method was employed with the following parameters: number of samples: 1000, 95% confidence intervals, bias-corrected accelerated.

Before logistic regression analysis, the underlying assumptions were verified, including the linear relationship between the independent variables and the log-odds of the dependent variable (rather than a direct linear relationship), and the absence of multicollinearity among independent variables (variance inflation factor <5). Variables that showed statistically significant differences in the preliminary hypothesis tests were included as covariates in the regression model. For comparisons between two groups, *P*-values were adjusted using the Bonferroni method. For comparisons among three groups, *P*-values were adjusted with the Welch test and the Brown–Forsythe test. In the case of multiple comparisons, if homogeneity of variance was satisfied, the Tukey test was used for *P*-value adjustment; otherwise, the Games–Howell test was applied.

## Results

### Prevalence of anxiety symptoms and its correlation with RSA

Among 1718 patients with MDD, 346 (20.1%) had a history of RSA. Compared with patients without an RSA, patients with a history of RSA had higher HRSD scores, PANSS positive subscale scores, FBG, total serum cholesterol, LDL-C, SBP and DBP; and abnormal rates of TSH, TgAb and TPOAb; and lower HDL-C levels (all *P* < 0.05). In this study, all patients with MDD had anxiety symptoms (HRSA score ≥14) except one patient without RSA who did not have anxiety symptoms (HRSA score <14). Among patients with a history of RSA, 62 had anxiety, 247 had significant anxiety and 37 had severe anxiety. Among patients without a history of RSA, 761 had anxiety, 602 had significant anxiety and eight had severe anxiety. Anxiety severity was significantly higher in patients with a history of RSA than in those without an RSA (*P* < 0.001). After adjusting for the disease duration, HRSD score, PANSS positive subscale score, FBG, total serum cholesterol, HDL-C, LDL-C, SBP, DBP and the abnormal status of TSH, TgAb and TPOAb, the binary logistic regression analysis revealed that the severity of anxiety was an independent related factor for RSA. The risk of RSA in patients with significant anxiety and severe anxiety was 2.9 times and 11.8 times higher than that in patients with anxiety, respectively.

### Differences in clinical factors among patients with different anxiety severity

**Fig. 2:**
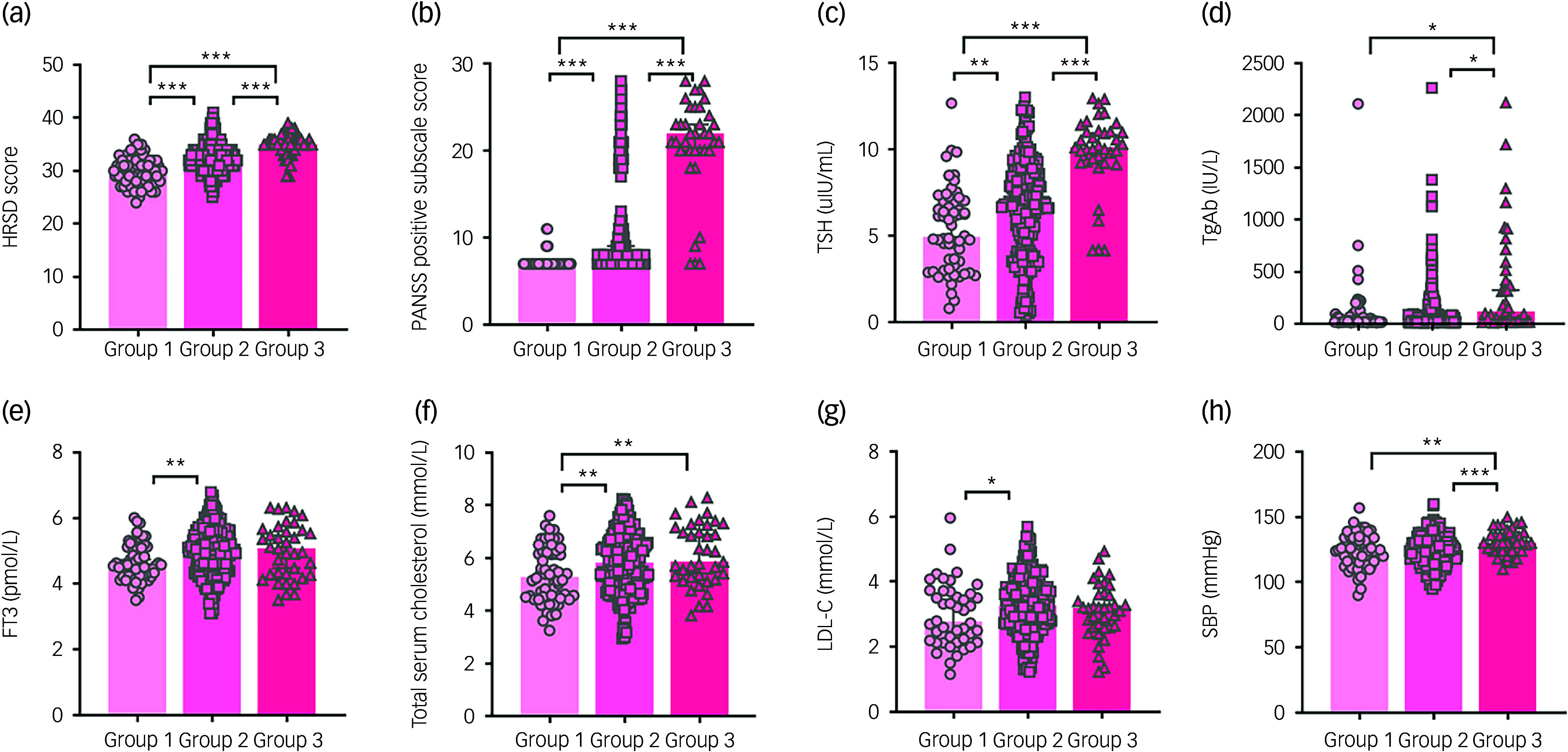
Differences in clinical characteristics with different anxiety severity. Group 1: patients with anxiety, group 2: patients with significant anxiety, group 3: patients with severe anxiety. Panels show differences in HRSD, PANSS positive subscale score, TSH, TgAb, FT3, total serum cholesterol, LDL-C and SBP with different anxiety severity. HRSD, Hamilton Rating Scale for Depression; PANSS, Positive and Negative Syndrome Scale; TSH, thyroid-stimulating hormone; TgAb, anti-thyroglobulin antibody; FT3, free triiodothyronine; LDL-C, low-density lipoprotein cholesterol; SBP, systolic blood pressure. *P < 0.05; **P < 0.01; ***P < 0.001.

As shown in Table 1, among patients with MDD and a history of RSA, there were significant differences in HRSD score (*F*(2,82) = 43.82, mean squared error (MSE) = 6.77, *P* < 0.001, *r* = 0.44), PANSS positive subscale score (*F*(2,87) = 145.13, MSE = 29.88, *P* < 0.001, *r* = 0.54), TSH (*F*(2,86) = 45.33, MSE = 7.10, *P* < 0.001, *r* = 0.40), TgAb (*F*(2,69) = 4.21, MSE = 84 819.73, *P* < 0.05, *r* = 0.26), FT3 (*F*(2,82) = 6.00, MSE = 0.52, *P* < 0.01, *r* = 0.15), total serum cholesterol (*F*(2,79) = 7.51, MSE = 1.18, *P* < 0.01, *r* = 0.20), LDL-C (*F*(2,79) = 3.22, MSE = 0.81, *P* < 0.05, *r* = 0.14) and SBP (*F*(2,83) = 9.72, MSE = 138.47, *P* < 0.001, *r* = 0.20) among those with different anxiety severity. The specific differences between groups are shown in Table 2 and Fig. 2.

**Table 1:** Clinical characteristics in patients with major depressive disorder and recent suicide attempts, by anxiety severity

Variables	Total	Group			Effect size (*r*)	Corrected *P*-value
		Patients with MDD and RSA, with anxiety	Patients with MDD and RSA, with significant anxiety	Patients with MDD and RSA, with severe anxiety		
*n*	346	62	247	37		
HRSD score	32.24 ± 2.89	30.11 ± 2.76	32.37 ± 2.61	34.92 ± 2.27	0.44	<0.001***
PANSS positive subscale score	11.48 ± 6.49	7.22 ± 0.82	11.17 ± 6.06	20.68 ± 5.71	0.54	<0.001***
TSH, uIU/mL	6.71 ± 2.91	5.36 ± 2.41	6.59 ± 2.78	9.83 ± 2.24	0.40	<0.001***
TgAb, IU/L	148.58 ± 300.59	114.19 ± 289.05	123.69 ± 241.97	372.37 ± 516.21	0.26	0.02*
FT3, pmol/L	4.91 ± 0.73	4.68 ± 0.56	4.96 ± 0.74	5.00 ± 0.83	0.15	0.004**
Total serum cholesterol, mmol/L	5.77 ± 1.11	5.34 ± 1.03	5.83 ± 1.08	6.12 ± 1.18	0.20	0.001**
LDL-C, mmol/L	3.21 ± 0.91	2.96 ± 0.98	3.29 ± 0.89	3.11 ± 0.84	0.14	0.045*
SBP, mmHg	124.40 ± 11.97	123.32 ± 13.11	123.65 ± 11.70	131.24 ± 9.67	0.20	<0.001***

MDD, major depressive disorder; RSA, recent suicide attempts; HRSD, Hamilton Rating Scale for Depression; PANSS, Positive and Negative Syndrome Scale; TSH, thyroid-stimulating hormone; TgAb, anti-thyroglobulin antibody; FT3, free triiodothyronine; LDL-C, low-density lipoprotein cholesterol; SBP, systolic blood pressure.

*P < 0.05; **P < 0.01; ***P < 0.001.

**Table 2:** Differences of clinical characteristics in anxiety of different severity

Variables	Group		*t*	*d*	Corrected *P*-value	95% CI
HRSD score	Severe anxiety	Anxiety	8.91	1.85	<0.001***	(3.53−6.08)
		Significant anxiety	5.56	0.98	<0.001***	(1.47−3.63)
	Significant anxiety	Anxiety	6.11	0.87	<0.001***	(1.39−3.13)
PANSS positive subscale score	Severe anxiety	Anxiety	11.80	2.46	<0.001***	(11.14−15.76)
		Significant anxiety	9.91	1.74	<0.001***	(7.05−11.96)
	Significant anxiety	Anxiety	5.05	0.72	<0.001***	(3.00−4.89)
TSH	Severe anxiety	Anxiety	8.13	1.68	<0.001***	(3.33−5.62)
		Significant anxiety	6.89	1.22	<0.001***	(2.26−4.23)
	Significant anxiety	Anxiety	3.24	0.46	0.002**	(0.39−2.07)
TgAb	Severe anxiety	Anxiety	4.27	0.89	0.02*	(34.79−481.56)
		Significant anxiety	4.84	0.85	0.02*	(38.42−458.94)
	Significant anxiety	Anxiety	0.23	0.03	0.97	(−85.49 to 104.48)
FT3	Severe anxiety	Anxiety	2.20	0.46	0.09	(−0.04 to 0.70)
		Significant anxiety	0.31	0.06	0.95	(−0.31 to 0.39)
	Significant anxiety	Anxiety	2.73	0.39	0.003**	(0.08−0.49)
Total serum cholesterol	Severe anxiety	Anxiety	3.50	0.73	0.002**	(0.26−1.32)
		Significant anxiety	1.51	0.27	0.29	(−0.16 to 0.74)
	Significant anxiety	Anxiety	3.24	0.46	0.004**	(0.13−0.86)
LDL-C	Severe anxiety	Anxiety	0.86	0.18	0.68	(−0.28 to 0.60)
		Significant anxiety	−1.07	−0.19	0.52	(−0.55 to 0.20)
	Significant anxiety	Anxiety	2.58	0.37	0.03*	(0.03−0.63)
SBP	Severe anxiety	Anxiety	3.24	0.67	0.004**	(2.17−13.68)
		Significant anxiety	3.66	0.65	<0.001***	(2.71−12.48)
	Significant anxiety	Anxiety	0.20	0.03	0.98	(−3.61 to 4.26)

HRSD, Hamilton Rating Scale for Depression; PANSS, Positive and Negative Syndrome Scale; TSH, thyroid-stimulating hormone; TgAb, anti-thyroglobulin antibody; FT3, free triiodothyronine; LDL-C, low-density lipoprotein cholesterol; SBP, systolic blood pressure.

*P < 0.05; **P < 0.01; ***P < 0.001.

### Effect of clinical characteristics on anxiety severity in patients with MDD and a history of RSA

Table 3 showed the extent to which different factors were associated with anxiety severity in patients with MDD and a history of RSA. Multivariate logistic regression analysis revealed that, among these patients, HRSD score and PANSS positive subscale score were independent related factors for distinguishing between levels of anxiety severity (anxiety, significant anxiety and severe anxiety). FT3 emerged as an independent related factor for differentiating anxiety from both significant and severe anxiety, whereas TSH was identified as an independent related factor for distinguishing between anxiety and significant anxiety collectively versus severe anxiety.

**Table 3:** Effect of clinical characteristics on anxiety severity in patients with major depressive disorder and a history of recent suicide attempts

Variables	Group		*B*	s.e.	*P*-value	Odds ratio	95% CI
HRSD score	Severe anxiety	Anxiety	0.42	0.13	<0.001***	1.53	(1.19−1.95)
		Significant anxiety	0.24	0.10	0.02*	1.28	(1.04−1.57)
	Significant anxiety	Anxiety	0.18	0.07	0.02*	1.20	(1.03−1.38)
PANSS positive subscale score	Severe anxiety	Anxiety	0.61	0.20	0.003**	1.83	(1.23−2.72)
		Significant anxiety	0.12	0.04	0.008**	1.12	(1.03−1.22)
	Significant anxiety	Anxiety	0.49	0.20	0.01*	1.63	(1.11−2.41)
TSH	Severe anxiety	Anxiety	0.38	0.14	0.01**	1.46	(1.11−1.92)
		Significant anxiety	0.36	0.11	0.002**	1.43	(1.14−1.79)
	Significant anxiety	Anxiety	0.02	0.08	0.84	1.02	(0.86−1.20)
TgAb	Severe anxiety	Anxiety	0	0	0.83	1.00	(1.00−1.00)
		Significant anxiety	0	0	0.12	1.00	(1.00−1.00)
	Significant anxiety	Anxiety	0	0	0.21	1.00	(1.00−1.00)
FT3	Severe anxiety	Anxiety	0.96	0.39	0.02*	2.60	(1.20−5.62)
		Significant anxiety	0.34	0.32	0.29	1.41	(0.75−2.64)
	Significant anxiety	Anxiety	0.62	0.23	0.01**	1.85	(1.17−2.92)
Total serum cholesterol	Severe anxiety	Anxiety	−0.10	0.31	0.74	0.90	(0.49−1.65)
		Significant anxiety	−0.19	0.23	0.42	0.83	(0.53−1.30)
	Significant anxiety	Anxiety	0.08	0.21	0.70	1.09	(0.72−1.64)
LDL-C	Severe anxiety	Anxiety	−0.43	0.38	0.25	0.65	(0.31−1.36)
		Significant anxiety	−0.59	0.31	0.05	0.55	(0.30−1.01)
	Significant anxiety	Anxiety	0.16	0.23	0.48	1.18	(0.75−1.83)
SBP	Severe anxiety	Anxiety	0	0.03	0.88	1.00	(0.95−1.06)
		Significant anxiety	0.027	0.02	0.25	1.03	(0.98−1.08)
	Significant anxiety	Anxiety	−0.023	0.01	0.10	0.98	(0.95−1.01)

HRSD, Hamilton Rating Scale for Depression; PANSS, Positive and Negative Syndrome Scale; TSH, thyroid-stimulating hormone; TgAb, anti-thyroglobulin antibody; FT3, free triiodothyronine; LDL-C, low-density lipoprotein cholesterol; SBP, systolic blood pressure.

*P < 0.05; **P < 0.01; ***P < 0.001.

## Discussion

The main findings of this study are as follows. First, anxiety severity demonstrated an independent positive association with risk of RSA in patients with MDD; compared with patients with MDD and mild anxiety, those with significant anxiety and severe anxiety had 2.9 times and 11.8 times higher risks of RSA, respectively. Second, among patients with MDD and a history of RSA, higher levels of depressive symptoms, positive psychosis symptoms and thyroid function indicators (FT3 and TSH) are independently associated with increased anxiety severity.

This study assessed anxiety symptoms in 1718 patients with MDD, using the HRSA scale, with 1717 patients evaluated as having varying degrees of anxiety, indicating an extremely high comorbidity rate of anxiety in the MDD population. However, this finding raises an important question: whether the HRSA scale may be overly sensitive in assessing anxiety, potentially leading to an overestimation of comorbid anxiety in patients with depression. The result profoundly highlights the methodological limitations of relying on a single symptom scale in MDD: the high sensitivity of the HRSA primarily stems from its inherent overlap with core MDD symptoms (such as psychic anxiety, insomnia and somatic discomfort), which compromises its specificity. Consequently, what is being measured is essentially the anxiety symptom dimension within MDD, rather than a truly independent comorbid condition. The resulting ceiling effect homogenises the sample in terms of the anxiety variable, substantially weakening the scale’s discriminant validity and the precision of treatment outcome evaluation. Moreover, the pervasiveness of anxiety symptoms suggests that they may be an intrinsic component of the MDD disease phenotype, thereby diluting the clinical significance of stratifying patients or conducting association studies based solely on HRSA scores. Therefore, this phenomenon underscores the necessity, in complex psychopathology research, of employing a multi-faceted approach – integrating clinical interviews, multidimensional scales and factor analysis – to more accurately delineate the clinical meaning of symptom structures.

Research on the correlation between anxiety and suicide risk has yielded mixed results. For example, one study indicated that individuals with anxiety disorders were five times more likely to attempt suicide compared with those without.^16^ In contrast, other studies have found no significant association between anxiety and suicidal behaviour, and some have even reported the opposite, suggesting that anxiety may be linked to a lower risk of suicide.^17^ The discrepancies across these findings may be attributed to factors such as methodological variations, differences in inclusion/exclusion criteria and sample heterogeneity. Furthermore, in some previous studies, a significant temporal gap between the assessment of anxiety levels and the occurrence of suicidal behaviour may weaken the observed correlation between the current state of anxiety and suicide risk. It is also important to note that although both suicidal ideation and suicidal behaviour are categorised under suicide risk, they represent distinct constructs. Core principles of the ideation-to-action framework suggest that the transition from having no suicidal ideation to developing ideation, and from ideation to engaging in suicidal behaviour, are distinct processes with different underlying mechanisms.^18^ In this study, the severity of anxiety in patients with MDD and a history of suicide attempts within the past month was assessed with the HRSA. We found that the severity of anxiety symptoms was significantly associated with suicide risk in patients with MDD. Specifically, patients with MDD and severe anxiety exhibited a more than ten-fold higher suicide risk compared with those with mild anxiety. The mechanisms underlying the influence of anxiety on suicide are complex. Anxiety sensitivity may play a role in mediating the impact of anxiety on suicidal behaviour.^19^ Research has confirmed an association between anxiety sensitivity and suicide, with significantly elevated anxiety sensitivity levels observed in individuals who have attempted suicide.^20^ Anxiety sensitivity may exacerbate the experience of anxiety and, in patients at high risk of suicide, could facilitate the progression from suicidal ideation to suicidal behaviour.^21^ Furthermore, it may also influence suicide risk through indirect pathways. For instance, its role in promoting maladaptive coping strategies can increase the likelihood of developing suicidal ideation.^22–24^

Although this study cannot establish a causal relationship between depression severity and anxiety severity, their significant positive correlation has been consistently demonstrated here and in numerous other studies, highlighting the need for increased clinical attention to this comorbidity. This association may stem from shared neurobiological underpinnings interacting with environmental stressors, leading the two disorders to frequently influence and exacerbate each other.^25,26^ Furthermore, our findings revealed a positive correlation between positive psychotic symptoms and anxiety severity, independent of depressive symptoms. This aligns with observations by Temmingh et al in schizophrenia populations.^27^ The mechanisms underlying this link may involve multiple pathways, with sleep disturbances potentially serving as a mediating factor. Evidence suggests that sleep impairment is closely associated with the worsening of positive symptoms and can significantly intensify anxiety.^28^ Therefore, future studies should include sleep quality as a key factor in both clinical evaluation and intervention strategies, to better understand its specific contribution to the link between positive symptoms and anxiety severity.

Additionally, the association between TSH levels and anxiety severity observed in the present study is consistent with earlier findings by Zhu et al in patients with MDD, further supporting the hypothesis that thyroid axis function may be linked to mood disorders.^26^ From a neurobiological perspective, it has been proposed that thyroid hormones might influence the plasticity of key emotional regulatory brain regions such as the hippocampus, thereby potentially contributing to the pathophysiology of anxiety. Specifically, thyroid hormones are known to modulate the expression of neurotrophic factors, including brain-derived neurotrophic factor (BDNF), which plays a critical role in neuronal survival, differentiation and synaptic plasticity.^29^ Thus, thyroid dysfunction may indirectly affect the functional integration and adaptive changes within emotional neural networks by regulating BDNF-related signalling pathways, possibly exacerbating or sustaining anxiety symptoms. It is noteworthy that the aforementioned mechanistic explanation remains speculative and has not yet been directly validated in populations with anxiety disorders. Current understanding is largely derived from animal models and neurophysiological studies in individuals with thyroid diseases, and the specific pathways and modes of action in human anxiety disorders require further investigation through longitudinal and mechanistic research. Future studies should integrate multi-modal neuroimaging, dynamic monitoring of peripheral biomarkers and genetic tools to more systematically explore the role of thyroid–brain interactions in the development and progression of anxiety disorders. Such approaches could provide a more solid foundation for understanding disease mechanisms and potential intervention strategies.

Our findings support a close association between anxiety and depressive symptoms, suggesting that in the clinical management of MDD, the assessment of anxiety severity should not be viewed merely as a descriptor of comorbidity, but should be integrated as a key dimension in core risk stratification and treatment decision-making. Elevated anxiety levels may identify a more complex and vulnerable MDD subgroup, characterised by more severe depressive symptoms, greater functional impairment, more prominent sleep and somatic complaints, and a potentially poorer initial treatment response. Therefore, systematic screening using conventional depression assessments alongside targeted anxiety scales, such as the Generalised Anxiety Disorder scale, can facilitate more precise disease stratification and prognosis estimation. In terms of management, identifying and actively addressing significant anxiety is crucial for optimising overall treatment strategies. Evidence indicates that patients with MDD and severe anxiety may require more nuanced therapeutic approaches; for instance, selecting antidepressants with efficacy across anxiety spectrum symptoms while considering potential activating side-effects, and integrating anxiety-focused cognitive–behavioural or mindfulness-based techniques within psychotherapy to address emotional distress more comprehensively. Furthermore, the observed links among anxiety, positive psychotic symptoms and neuroendocrine indicators such as TSH underscore the necessity of a transdiagnostic, multi-system perspective. Clinicians managing patients with MDD and prominent anxiety should remain alert to broader psychopathological network activity, including the assessment of psychotic symptoms and consideration of physiological tests such as thyroid function. In summary, dynamically incorporating anxiety severity into the holistic assessment and management framework for MDD not only promotes more individualised and proactive care but also provides a clinical foundation for shifting from a symptom-specific treatment model toward dimension-based transdiagnostic intervention. Future studies should further clarify the long-term outcomes of the high-anxiety MDD subtype and validate the effectiveness of integrated intervention strategies in this population.

This study has several limitations that should be noted. First, the cross-sectional design restricts causal interpretations regarding anxiety and RSA, as well as the relationships between other clinical factors and anxiety. Second, the demographic characteristics and the observed prevalence of anxiety in this study may be influenced by cultural or sampling factors. For instance, nearly all participants in this study exhibited mild anxiety symptoms, and very few were without anxiety, which may limit the generalisability of conclusions regarding the extent of anxiety’s role in RSA. Finally, suicide risk was assessed through face-to-face interviews in this study; the use of structured suicide risk assessment scales could help reduce potential bias in such evaluations.

In summary, our findings highlight an independent positive association between anxiety severity and RSA in patients with MDD, emphasising the clinical importance of managing anxiety symptoms in this population. This study also reveals independent correlations of anxiety with depressive symptoms, positive psychotic symptoms and thyroid hormone levels. Future research should systematically examine the differential effects of distinct dimensions of anxiety symptoms, such as psychic anxiety and somatic anxiety, as well as specific anxiety-related behaviours, including avoidance and hypervigilance, on the risk of suicide attempts in MDD. Such investigations will not only help clarify the specific pathways through which anxiety contributes to suicidal behaviour in MDD, but also provide an empirical basis for developing precise early-warning indicators and targeted symptom-specific interventions for high-risk individuals, thereby supporting the implementation of more stratified and personalised comprehensive treatment strategies in clinical practice.

## Data Availability

Access to the raw data supporting the findings of this study can be obtained from the corresponding author upon reasonable request and in accordance with ethical protocols.

## References

[1] Borentain S , Nash AI , Dayal R , DiBernardo A. Patient-reported outcomes in major depressive disorder with suicidal ideation: a real-world data analysis using PatientsLikeMe platform. BMC Psychiatry 2020; 20: 384. 32703173 10.1186/s12888-020-02758-y PMC7376651

[2] Hawton K , Casañas i Comabella C , Haw C , Saunders K. Risk factors for suicide in individuals with depression: a systematic review. J Affect Disord 2013; 147: 17–28. 23411024 10.1016/j.jad.2013.01.004

[3] Moriya J , Tanno Y. Dysfunction of attentional networks for non-emotional processing in negative affect. Cogn Emot 2009; 23: 1090–105.

[4] Tarsia M , Power MJ , Sanavio E. Implicit and explicit memory biases in mixed anxiety-depression. J Affect Disord 2003; 77: 213–25. 14612221 10.1016/s0165-0327(02)00119-2

[5] Basso MR , Lowery N , Ghormley C , Combs D , Purdie R , Neel J , et al. Comorbid anxiety corresponds with neuropsychological dysfunction in unipolar depression. Cogn Neuropsychiatry 2007; 12: 437–56. 17691001 10.1080/13546800701446517

[6] Castaneda AE , Marttunen M , Suvisaari J , Perälä J , Saarni SI , Aalto-Setälä T , et al. The effect of psychiatric co-morbidity on cognitive functioning in a population-based sample of depressed young adults. Psychol Med 2010; 40: 29–39. 19413917 10.1017/S0033291709005959

[7] So H-C , Chau CK-L , Cheng Y-Y , Sham PC. Causal relationships between blood lipids and depression phenotypes: a Mendelian randomisation analysis. Psychol Med 2020; 51: 2357–69. 32329708 10.1017/S0033291720000951

[8] Pan LA , Naviaux JC , Wang L , Li K , Monk JM , Lingampelly SS , et al. Metabolic features of treatment-refractory major depressive disorder with suicidal ideation. Transl Psychiatry 2023; 13: 393. 38097555 10.1038/s41398-023-02696-9 PMC10721812

[9] Baek JH , Kang E-S , Fava M , Mischoulon D , Nierenberg AA , Yu B-H , et al. Serum lipids, recent suicide attempt and recent suicide status in patients with major depressive disorder. Prog Neuropsychopharmacol Biol Psychiatry 2014; 51: 113–8. 24495778 10.1016/j.pnpbp.2014.01.018

[10] Zhang Q , Zhao S , Liu Z , Luo B , Yang Y , Shi Y , et al. Association of thyroid-stimulating hormone and lipid levels with suicide attempts among adolescents with major depressive disorder in China. Front Psychiatry 2023; 13: 1031945. 36733417 10.3389/fpsyt.2022.1031945 PMC9887045

[11] Zhao S , Wang A , Han Y , Liu Y , Fang W , Cheng X , et al. Dynamic brain activity and thyroid dysregulation in suicidal ideation of MDD. J Affect Disord 2025; 392: 120195. 40907715 10.1016/j.jad.2025.120195

[12] Sun XY , Li YX , Yu CQ , Li LM. Reliability and validity of depression scales of Chinese version: a systematic review. Zhonghua Liu Xing Bing Xue Za Zhi 2017; 38: 110–6. 28100388 10.3760/cma.j.issn.0254-6450.2017.01.021

[13] Lin GX. Uses of HAMA the rating scale in neurosis. Zhonghua Shen Jing Jing Shen Ke Za Zhi 1986; 19: 342–4. 3582026

[14] Wu B-J , Lan T-H , Hu T-M , Lee S-M , Liou J-Y. Validation of a five-factor model of a Chinese Mandarin version of the Positive and Negative Syndrome Scale (CMV-PANSS) in a sample of 813 schizophrenia patients. Schizophr Res 2015; 169: 489–90. 26443481 10.1016/j.schres.2015.09.011

[15] Zhang M , He Y. Handbook of Psychiatric Rating Scales. Hunan Science and Technology Press, 2015.

[16] Moitra M , Santomauro D , Degenhardt L , Collins PY , Whiteford H , Vos T , et al. Estimating the risk of suicide associated with mental disorders: a systematic review and meta-regression analysis. J Psychiatr Res 2021; 137: 242–9. 33714076 10.1016/j.jpsychires.2021.02.053 PMC8095367

[17] Nock MK , Hwang I , Sampson NA , Kessler RC. Mental disorders, comorbidity and suicidal behavior: results from the National Comorbidity Survey Replication. Mol Psychiatry 2010; 15: 868–76. 19337207 10.1038/mp.2009.29 PMC2889009

[18] Klonsky ED , Dixon-Luinenburg T , May AM. The critical distinction between suicidal ideation and suicide attempts. World Psychiatry 2021; 20: 439–41. 34505359 10.1002/wps.20909 PMC8429339

[19] Stanley IH , Boffa JW , Rogers ML , Hom MA , Albanese BJ , Chu C , et al. Anxiety sensitivity and suicidal ideation/suicide risk: a meta-analysis. J Consult Clin Psychol 2018; 86: 946–60. 30335426 10.1037/ccp0000342 PMC6469498

[20] Demirkol ME , Tamam L , Namli Z , Karaytuğ MO , Yeşiloğlu C. The relationship among anxiety sensitivity, psychache, and suicidality in patients with generalized anxiety disorder. J Nerv Ment Dis 2022; 210: 760–6. 35605224 10.1097/NMD.0000000000001534

[21] Allan NP , Gorka SM , Saulnier KG , Bryan CJ. Anxiety sensitivity and intolerance of uncertainty: transdiagnostic risk factors for anxiety as targets to reduce risk of suicide. Curr Psychiatry Rep 2023; 25: 139–47. 37000403 10.1007/s11920-023-01413-z PMC10064604

[22] Poorolajal J , Haghtalab T , Farhadi M , Darvishi N. Substance use disorder and risk of suicidal ideation, suicide attempt and suicide death: a meta-analysis. J Public Health (Oxf) 2016; 38: e282–91. 26503486 10.1093/pubmed/fdv148

[23] Allan NP , Albanese BJ , Norr AM , Zvolensky MJ , Schmidt NB. Effects of anxiety sensitivity on alcohol problems: evaluating chained mediation through generalized anxiety, depression and drinking motives. Addiction 2015; 110: 260–8. 25220033 10.1111/add.12739 PMC4302006

[24] DeMartini KS , Carey KB. The role of anxiety sensitivity and drinking motives in predicting alcohol use: a critical review. Clin Psychol Rev 2011; 31: 169–77. 21074306 10.1016/j.cpr.2010.10.001

[25] Coussement C , De Longueville X , Heeren A. Attentional networks in co-occurring generalized anxiety disorder and major depression disorder: towards a staging approach to the executive control deficits. Compr Psychiatry 2022; 113: 152294. 34942482 10.1016/j.comppsych.2021.152294

[26] Zhu Y , Yin W , Ma J , Zhang L. Anxious depression in major depressive disorder: key influences and prevalence in Chinese hospitalized patients. Neuropsychiatr Dis Treat 2024; 20: 2267–75. 39619492 10.2147/NDT.S499392 PMC11606164

[27] Temmingh H , Stein DJ. Anxiety in patients with schizophrenia: epidemiology and management. CNS Drugs 2015; 29: 819–32. 26482261 10.1007/s40263-015-0282-7

[28] Blanchard JJ , Andrea A , Orth RD , Savage C , Bennett ME. Sleep disturbance and sleep-related impairment in psychotic disorders are related to both positive and negative symptoms. Psychiatry Res 2020; 286: 112857. 32087449 10.1016/j.psychres.2020.112857 PMC7416463

[29] Yu D , Zhou H , Yang Y , Jiang Y , Wang T , Lv L , et al. The bidirectional effects of hypothyroidism and hyperthyroidism on anxiety- and depression-like behaviors in rats. Horm Behav 2015; 69: 106–15. 25623236 10.1016/j.yhbeh.2015.01.003

